# Peripheral Gangrene Complicating Systemic Lupus Erythematosus in a Patient with Spina Bifida: A Case Report

**DOI:** 10.5704/MOJ.1703.009

**Published:** 2017-03

**Authors:** S Vijay, VK Imthiaz, S Hitesh

**Affiliations:** Department of Orthopaedics, Manipal University Kasturba Medical College, Karnataka, India

**Keywords:** spinal dysraphism, lupus erythematosus, gangrene, spina bifida, foot

## Abstract

An adolescent girl, a known case of spina bifida with systemic lupus, presented with bluish discolouration of three toes of the right foot. She had thrombosis of bilateral popliteal arteries. She underwent percutaneous transluminal angioplasty (PTA) of both legs and Chopart amputation of the right foot. Systemic lupus erythematosus (SLE) occurring in a patient with spina bifida has not been previously reported. Weakness, sensory loss, lack of normal ambulation, endarteritis, antiphospholipid antibody syndrome are common contributory factors for peripheral gangrene in patients with spina bifida with systemic lupus erythematosus.

## Introduction

Spina bifida is the most common birth defect of the central nervous system^1^. To the best of our knowledge, systemic lupus erythematosus (SLE) occurring in a patient with spina bifida has not been reported in literature. Peripheral gangrene occurring in a patient with SLE is uncommon^2^. We present this as a rare case of peripheral gangrene complicating SLE in a patient with spina bifida.

## Case Report

**Fig. 1:**
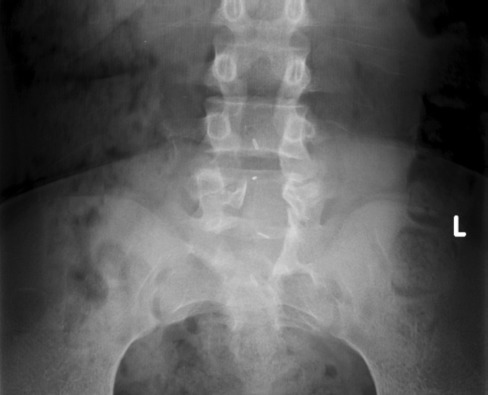
Anteroposterior view of lumbosacral spine showing L4-L5 spina bifida.

**Fig. 2:**
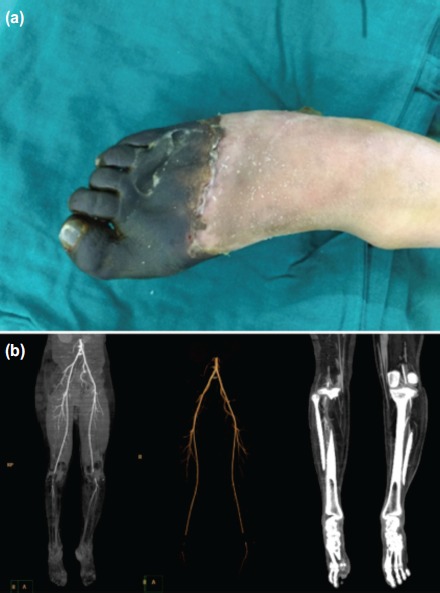
(a) Clinical picture of right foot showing gangrene of right forefoot and (b) CT angiogram of both lower limbs showing thrombosis of popliteal arteries.

On December 2015, a 19 years old girl presented to us with complaints of black discoloration of first, second and third toes of the right foot of 10 days duration. She was a known case of lower lumbar spina bifida who underwent lumbar myelomeningocele excision at three years of age (Fig. 1). She had bilateral flail ankles for which ankle foot orthosis had been prescribed and she was able to ambulate by herself. Her sensory and motor level was at L3. She had a neurogenic bladder and was on oxybutynin for the same. In 2009, at thirteen years of age, she developed fever with pain and swelling in both wrists and metacarpophalangeal joints. She was diagnosed to have SLE based on SLICC (Systemic Lupus International Collaborating Clinics) criteria. C3 complement level was 54 mg/dl, and C4 complement level was 5 mg/dl. Her ANA was positive (1:320) with a homogenous pattern. Anti-ds DNA was positive, and Rib-P protein was strongly positive. Anti-CCP, renal function tests, and urine protein analysis were normal. She was started on oral steroids. In 2010 after a year of treatment she improved symptomatically but since she had recurrent urinary tract infections, dimercaptosuucinic acid (DMSA) scan was done which was normal. Urine protein analysis showed nonnephrotic range proteinuria. She underwent renal biopsy which showed class III lupus nephritis (International Society of Nephrology criteria) and was started on pulsed cyclophosphamide therapy along with steroids, azathioprine, and hydroxychloroquine (HCQ). She completed cyclophosphamide therapy nine months earlier. She was currently on oral steroids and azathioprine. On present examination, she had bluish to black discolouration of the toes of the right foot. Dorsalis pedis and posterior tibial arteries pulsations were feeble on the right foot compared to the left, and the saturation was absent in the toes of the right foot. The gangrene progressed to involve the entire right forefoot (Fig. 2a). Three days later the patient started developing bluish discolouration of the third toe of her left foot.

Laboratory investigations revealed ESR at 121 mm/hr, CRP 164.9 and haemoglobin- 6.5mg/dl. Total cell count, platelets, and coagulation profile were within normal limits. Renal function tests and urine protein analysis were normal. Indirect immunofluorescence test showed positive ANA (1:320) with a homogenous pattern; Anti ds DNA was positive with a titre of 800 IU/ml. C3 complement level was 73, and C4 complement level was 11. Antibodies to Cardiolipin (IgG and IgM) and phospholipase A2 were negative. Plain radiographs of feet and leg were normal. Arterial Doppler showed monophasic waveforms in both arteries. CT angiography of both lower limbs showed thrombosis of popliteal arteries with reformation below trifurcation. In the right leg, there was no contrast opacification in anterior, posterior tibial and peroneal vessels. In the left leg, there was faint contrast opacification in all the three vessels (Fig. 2b).

**Fig. 3:**
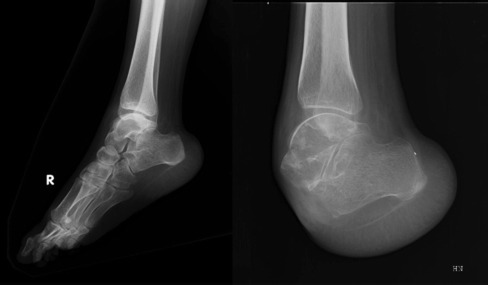
Preoperative and postoperative radiographs of right foot.

She was started on subcutaneous clexane and oral ecospirin. Oral steroids, azathioprine and HCQ, were continued. She underwent bilateral percutaneous transluminal angioplasty (PTA), and stents were placed in both popliteal arteries. A week later she underwent right foot Chopart amputation (Fig. 3). At one year follow-up, she was ambulant with left side ankle foot orthosis and right side ankle foot orthosis with a shoe.

Informed consent had been obtained at all stages of management.

## Discussion

Spina bifida is the most common birth defect that is compatible with survival^1^. The exact etiology of the spina bifida is unknown; however, it results from genetic and environmental factors, like folic acid deficiency and exposure to carbamazepine. Children with spina bifida present with weakness, muscle imbalance, decreased sensations, and bowel and bladder incontinence depending on the level of involvement^3^. Muscle imbalance usually leads to deformities. From an orthopaedic point of view, the prime aim involves correction of deformities that may prevent the patient from using orthoses during childhood^4^. Management of these patients requires a multidisciplinary team involving orthopaedic, neurosurgery, urology and physiotherapy. Some of the complications in these patients include latex allergy, pressure sores, recurrent urinary tract infection and pathologic fractures^3^. SLE is an autoimmune disease with unknown etiology. Joint swelling, fatigue and joint pain are characteristics of SLE. Complications like vasculitis, blood clots, nephrotic failure, pleuritis, and peripheral gangrene are reported.

We present this case for its unusual association between two different disabling pathologies. There are no reports in the literature of SLE occurring in a patient with spina bifida. Our patient had a lower lumbar spina bifida with sensory and motor level at L3. She had bilateral flail ankles and neurogenic bladder. She was diagnosed to have SLE at thirteen years of age. Drugs used in spina bifida were mainly for the treatment of neurogenic bladder which could not have caused SLE as symptoms should resolve once the offending agent is stopped.

The most common cause of extremity gangrene in SLE is an antiphospholipid antibody syndrome and endarteritis ^5^. Stasis, poor ambulation, vasculitis and blood clot might be contributing to the popliteal artery thrombosis in our patient. The main cause of gangrene was not only endarteritis, as the response to steroids and azathioprine was poor in this patient. She also developed ischemic changes in her left foot following the demarcation of gangrene in her right foot. She underwent bilateral percutaneous transluminal angioplasty, following which there was no progression of discoloration in the left foot. This highlights the importance of observation of contralateral limb in these patients for the development of peripheral gangrene. Our patient had bilateral flail ankles and absent sensation in both feet, which further complicated the treatment. We proceeded with Chopart amputation of the right foot.

In summary, weakness, sensory loss, lack of normal ambulation, endarteritis, and antiphospholipid antibody are common contributory factors for peripheral gangrene in patients with spina bifida with systemic lupus erythematosus. The physician must take these variables into consideration while managing the patient with SLE and spina bifida.
